# Stinging Nettle (*Urtica dioica *L.) Attenuates FFA Induced Ceramide Accumulation in 3T3-L1 Adipocytes in an Adiponectin Dependent Manner

**DOI:** 10.1371/journal.pone.0150252

**Published:** 2016-03-03

**Authors:** Diana N. Obanda, Peng Zhao, Allison J. Richard, David Ribnicky, William T. Cefalu, Jacqueline M. Stephens

**Affiliations:** 1 Pennington Biomedical Research Center, Louisiana State University System, Baton Rouge, Louisiana, United States of America; 2 Department of Plant Biology, Rutgers University, New Brunswick, New Jersey, United States of America; Université PARIS- DIDEROT (7), FRANCE

## Abstract

**Objective:**

Excess dietary lipids result in the accumulation of lipid metabolites including ceramides that can attenuate insulin signaling. There is evidence that a botanical extract of *Urtica dioica* L. (stinging nettle) improves insulin action, yet the precise mechanism(s) are not known. Hence, we examined the effects of *Urtica dioica* L. (UT) on adipocytes.

**Research Design:**

We investigated the effects of an ethanolic extract of UT on free fatty acid (palmitic acid) induced inhibition of insulin-stimulated Akt serine phosphorylation and modulation of ceramidase expression in 3T3-L1 adipocytes. Adipocytes were exposed to excess FFAs in the presence or absence of UT. Effects on adiponectin expression, ceramidase expression, ceramidase activity, ceramide accumulation and insulin signaling were determined.

**Results:**

As expected, FFAs reduced adiponectin expression and increased the expression of ceramidase enzymes but not their activity. FFA also induced the accumulation of ceramides and reduced insulin-stimulated phosphorylation of Akt in adipocytes. The effects of FFA were partially reversed by UT. UT enhanced adiponectin expression and ceramidase activity in the presence of excess FFAs. UT abated ceramide accumulation and increased insulin sensitivity via enhanced Akt phosphorylation. A siRNA knockdown of adiponectin expression prevented UT from exerting positive effects on ceramidase activity but not Akt phosphorylation.

**Conclusions:**

In adipocytes, the ability of UT to antagonize the negative effects of FFA by modulating ceramidase activity and ceramide accumulation is dependent on the presence of adiponectin. However, the ability of UT to enhance Akt phosphorylation is independent of adiponectin expression. These studies demonstrate direct effects of UT on adipocytes and suggest this botanical extract is metabolically beneficial.

## Introduction

Insulin resistance in skeletal muscle, liver and adipose tissue is strongly associated with metabolites that result from lipid oversupply in obesity. Studies have elucidated direct effects of the sphingolipid ceramide that mediates insulin resistance by maintaining Akt in an inactive dephosphorylated state [1–3]. Studies in rodent models of insulin resistance, diabetes, hepatic steatosis and atherosclerosis reveal that reducing the accumulation of ceramides delays or prevents disease onset [3].

Although difficult to achieve, lifestyle changes in nutrition and physical activity have been shown to be effective and desirable in reversing insulin resistance [4]. Botanical extracts that reduce pathogenic mechanisms involved in the etiology of insulin resistance represent an attractive complementary and alternative (CAM) approach for its prevention or treatment. Plants have traditionally been a rich source of medicinal compounds for many conditions, including metformin as a treatment for diabetes. *Urtica dioica* L. (UT), or stinging nettle, belongs to the family Urticaceae that includes more than 500 species worldwide. UT grows abundantly in northern Europe, Asia and Africa. In North America it is found in Canada and in the United States where it is present in every province and state except Hawaii [5]. The single-stemmed perennial, has a long history of use as a folk medicine and as a food source in several Asian and North African cultures and in Latin America. In some cultures it is used as a food supplement or salad ingredient without reported side effects [5, 6]. The blood glucose lowering effects of *Urtica dioica* as a medicinal plant has been documented and reviewed [6]. In a human study and several animal studies, there is evidence that *Urtica dioica* significantly decreases blood glucose and complications of diabetes through both pancreatic and extra pancreatic pathways. The beneficial effects of UT include anti-hyperglycemic effects in glucose tolerance tests [7–9], enhancement of islet insulin secretion [10] and anti-inflammatory effects [11].

Based on these reports [6–11], the objective of our study was to evaluate the efficacy and mechanisms of action of an ethanolic extract of *Urtica dioica* in cultured adipocytes. Using 3T3-L1 adipocytes as a model system in which metabolic dysregulation is induced using excess FFAs (palmitic acid), we investigated the effects of UT on expression and secretion of the insulin sensitizing hormone adiponectin and its ability to modulate the effects of FFA treatment. Excess FFAs down regulate the expression of adiponectin [12] and lead to the accumulation of lipid metabolites, particularly, ceramides [1–3]. Ceramide and its metabolites are known to interfere with insulin signaling in insulin sensitive cells and tissues resulting in insulin resistance *in vivo* [1–3].

Our data demonstrates that UT enhances the expression of adiponectin despite the presence of excess FFAs and activates ceramidases; a class of enzymes that catabolize ceramide into less deleterious metabolites. We further show that the beneficial effects of UT on ceramidase activity are negated when adiponectin is not present. Yet, UT was able to enhance Akt phosphorylation even in the absence of adiponectin. Our data are consistent with a study showing that the activity of ceramidases and ceramide accumulation are dependent on adiponectin levels [3]. In summary, our studies demonstrate that UT has metabolically beneficial effects in adipocytes *in vitro*.

## Materials and Methods

### Preparation and source of plant extract

*Urtica dioica* L. (UT) was harvested in New Jersey as the total herb above the root mass and a voucher specimen was produced by the Botanical Research Center and deposited in the Chrysler Herbarium at Rutgers University. The plant was harvested on the property of David Ribnicky, one of the authors of the manuscript, by David Ribnicky himself, with his permission for the use of the plant material. No endangered or protected species were involved. Geographic coordinates of the botanical collection site are 40°36'03.9"N 74°40'30.1"W. The freeze-dried plant was stored at -20°C. For extraction, 3 kg of dried plant was heated with 15 liters of 80% ethanol (v/v) to 80°C for 2 h and allowed to continue to extract for an additional 10 hours at 20°C. The extract was filtered through cheesecloth to remove particulates and ethanol was removed by rotary evaporation to less than 1 liter of final extract. The aqueous extract was then freeze-dried from -48 to 20°C in a glass tray. The dried extract was scraped from the glass tray and homogenized using a mortar and pestle. For cell culture experiments, the dried extracts were solubilized in 100% dimethyl sulfoxide (DMSO) at a concentration that was 1000-fold higher than experimental concentrations and then diluted into the cell media. A final concentration of 1-10ug/ml was used to treat cells.

### Preparation of FFAs

Lipid-containing media was prepared by conjugating palmitic acid sodium salt (Sigma Aldrich, St Louis MO) with fatty acid free BSA (Sigma Aldrich, St Louis MO). Briefly, a 50mls stock solution of 1000uM palmitic acid in DMEM was prepared by dissolving 13.75mg palmitic acid in 600ul MQ water in a 90°C (heat block). While maintained at 90°C, the dissolved fatty acid was added to 50ml warm media (40°C) in 25ul increments while stirring rapidly with a magnetic stirrer. After all fatty acid had been added, the solution was sterile filtered (0.2um filter) and stored at 4°C.

### Cell culture

Murine 3T3-L1 preadipocytes, obtained from Dr. Green’s laboratory [13], were grown, maintained and induced to differentiate using a standard protocol [14]. Fully differentiated adipocytes were maintained in DMEM supplemented with 10% FBS until utilized for experimentation. To initiate treatment, media was changed to DMEM supplemented with 5% calf serum. Cells were maintained in this media with fresh media added on day 2. Beginning on day 0, adipocytes were treated with FFAs, UT or DMSO vehicle daily. On day 3 after 72 hours treatment, 1 ml media was collected from each culture dish and 1mM phenylmethylsulfonylfluoride (PMSF) was added to each sample. The remaining media was aspirated, and cell monolayers collected for western blotting, enzyme activity assays and ceramide quantification.

### Transfection experiments

DharmaFECT DUO reagent, non-targeting siRNA, or adiponectin siRNA (all from GE Dharmacon, Lafayette, CO) were pre-incubated with OptiMEM media (Life technologies Carlsbad, CA) according to the manufacturer’s instructions. Mature adipocytes were trypsinized. 1.16 X10^5^ cells/cm^2^ were re-plated in 12-well plates and transfected by adding transfection reagents. After 24 h, media was changed to DMEM with 10% FBS. FFAs and UT treatments were added 48 hours after transfection.

### Cell extracts preparation for protein analysis, gel electrophoresis and immunoblotting

Whole cell extracts were prepared by harvesting adipocyte monolayers in lysis buffer containing 10 mM Tris (pH 7.4), 150 mM NaCl, 1 mM EGTA, 1 mM EDTA, 1% Triton X-100, 0.5% Igepal CA-630, 1 mM PMSF, 1 mM pepstatin, 1ul aprotinin, 10 mM leupeptin, 1 mM 1, 10-phenanthroline, and 0.2 mM sodium vanadate. Adipocyte monolayers were scraped into the lysis buffer, subjected to one freeze-thaw cycle, and passed 3 times through a 20-gauge needle. The lysates were then centrifuged at 17, 500 x g for 10 minutes at 4°C. After removing the floating lipid layer, protein concentrations of the supernatants were determined by a BCA kit (Thermo Scientific, Rockford, IL) according to the manufacturer’s instructions. As previously described [15], typical immunoblotting procedures were performed, and results were visualized with horseradish peroxidase-conjugated secondary antibodies and enhanced chemiluminescence. Primary antibodies included anti-adiponectin antibody (Thermo Scientific, Rockford, IL), phospho-Akt Ser473, anti Akt, anti-acid ceramidase, anti-neutral ceramidase (Santa Cruz, Dallas, TX), anti β-Actin and anti-tubulin (Sigma Aldrich, St Louis MO). Anti-rabbit and anti-mouse IgG secondary antibodies were from Sigma Aldrich (St Louis, MO). Optical densities of all protein bands were determined using NIH image J software as previously described [16].

### Ceramidase enzyme activity

Ceramidase activity was determined in cell lysates by quantifying the sphingosine generated from ceramide deacylation according to a method previously described [3] with modifications. Briefly cell lysates (100ug protein equivalent) were separately incubated for 22 h at 37°C with fluorescent analogues of ceramide i.e N-[6-[(7-nitro-2-1,3-benzoxadiazol-4-yl)amino]hexanoyl ceramide (NBD ceramide). Sphingosine labelled C18:0 NBD-ceramide was used for neutral ceramidase activity and C12:0 NBD-ceramide for acid ceramidase activity (both from Avanti polar lipids, Alabaster, AL). NBD-ceramide (8 nmol) was dried under inert gas and resuspended in assay detergent (7.5% octyl-β- d-glucopyranoside, 5 mM cardiolipin, 1 mM diethylenetriaminepentaacetic acid, pH 7) to help solubilize the lipid, plus buffer (25 mM Bis-Tris, pH 6.5, 0.3% Nonidet P-40). To achieve different pH conditions, 25 mM Bis-Tris (pH 6.5, pH 7) and 25 mM ammonium acetate (pH 5) were used to modify the buffer pH. NBD sphingosine (Avanti polar lipids, Alabaster, AL) was used as the standard in quantifying amount of fluorescence labelled sphingosine formed in the cell lysates. A FlexStation 3 multi-mode microplate reader was used to quantify fluorescence.

### Quantification of ceramide

Lipids were extracted from cell lysates and total ceramides quantified by tandemn mass spectrometry (LCMS/MS) as previously described [1,2].

### Statistical analyses

Optical densities of protein bands for adiponectin, ceramidases, phospho-Akt (Ser473), and total Akt were determined using image J software [16] and normalized by β-actin or tubulin before analysis by ANOVA followed by Fisher's least significant difference (LSD) tests. Ceramidase activity and total ceramide quantities were also compared among treatments by ANOVA followed by Fisher's least significant difference (LSD) tests. All data are representative of three independent experiments.

## Results

### Effects of FFAs and *Urtica dioica* extract on adiponectin expression

**Fig 1:**
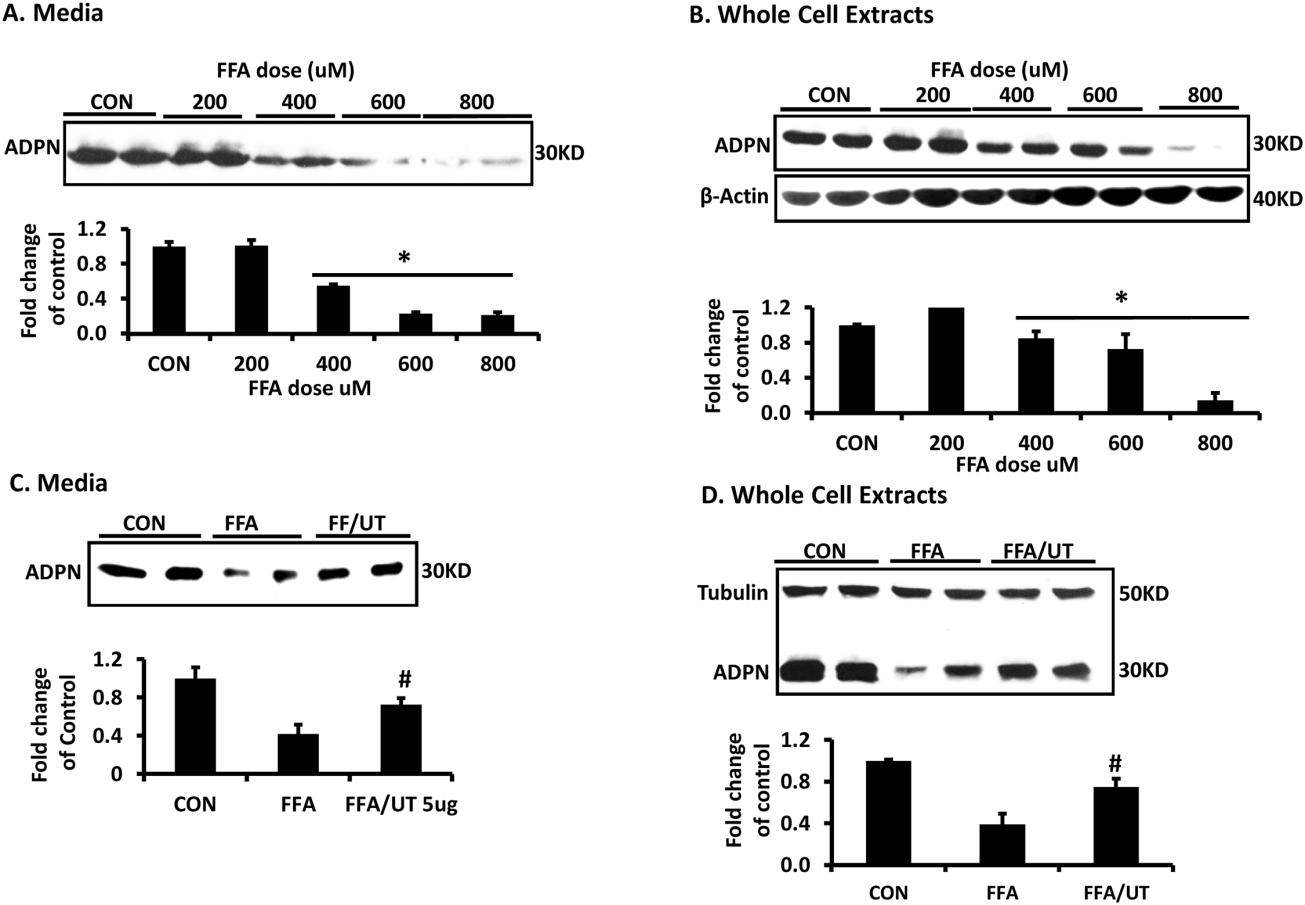
*Urtica dioica* extract partially rescues FFA induced reduction in adiponectin expression. Fully differentiated 3T3-L1 adipocytes were incubated with indicated doses of palmitate for 72 hours and media **(A)** and whole cell extracts **(B)** were examined for adiponectin expression. Adipocytes were also treated with palmitate for 72 hours in the presence or absence of UT (*Urtica dioica)* for 72 hours and media **(C)** and whole cell extracts **(D)** were examined for adiponectin expression. Samples were resolved by SDS-PAGE, transferred to nitrocellulose, and immunoblotted with the respective antibodies before detection by enhanced chemiluminescence. Each panel represents an experiment independently repeated three times on independent batches of adipocytes. ADPN expression in media and cells was normalized by the corresponding β-Actin expression in cells. Treatments were compared by ANOVA followed by the Tukeys test of multiple comparisons. *denotes significant difference between the control treatments and FFA treatment (P<0.05). ^**#**^denotes significant difference between the FFA and FFA + UT treatments (P<0.05)

As expected, exposure to FFAs for 72 hours significantly reduced adiponectin expression and secretion from 3T3-L1 adipocytes in a dose dependent manner (Fig 1A and 1B). Co-incubation with FFA and 5 ug/ml UT significantly attenuated the ability of FFAs to reduce adiponectin expression and secretion (P<0.05; Fig 1C and 1D).

### Effects of FFAs and *Urtica dioica* extract on ceramidase expression, activity and ceramide accumulation

**Fig 2:**
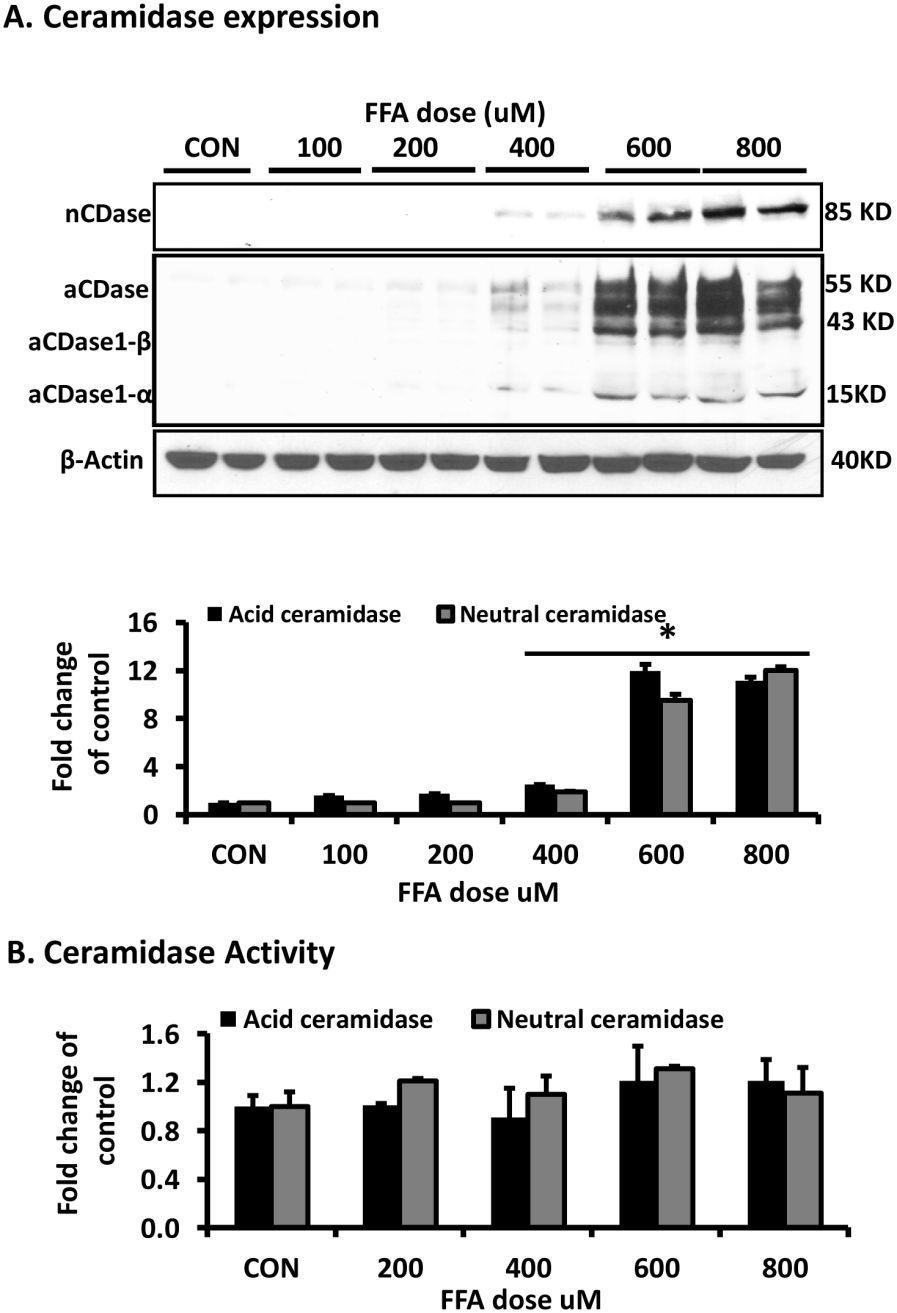
FFAs induce ceramidase protein expression in a dose dependent manner but do not increase ceramidase activity in adipocytes. Fully differentiated 3T3-L1 adipocytes were incubated with indicated doses of palmitate for 72 hours and **(A)** cell extracts were examined for acid ceramidase and neutral ceramidase expression. Samples were resolved by SDS-PAGE, transferred to nitrocellulose, and immunoblotted with the respective antibodies before detection by enhanced chemiluminescence. **(B)** Neutral ceramidase activity was determined by fluorometric quantification of the fluorescent sphingosine generated in the cells after 24 hours incubation with C18 NBD ceramide. Acid ceramidase was similarly determined after incubation with C12 NBD ceramide. Treatments were compared by ANOVA followed by Tukeys test of multiple comparisons. Each panel represents an experiment independently repeated three times on independent groups of adipocytes. *denotes significant difference between the control treatments and FFA treatment (P<0.05).

**Fig 3:**
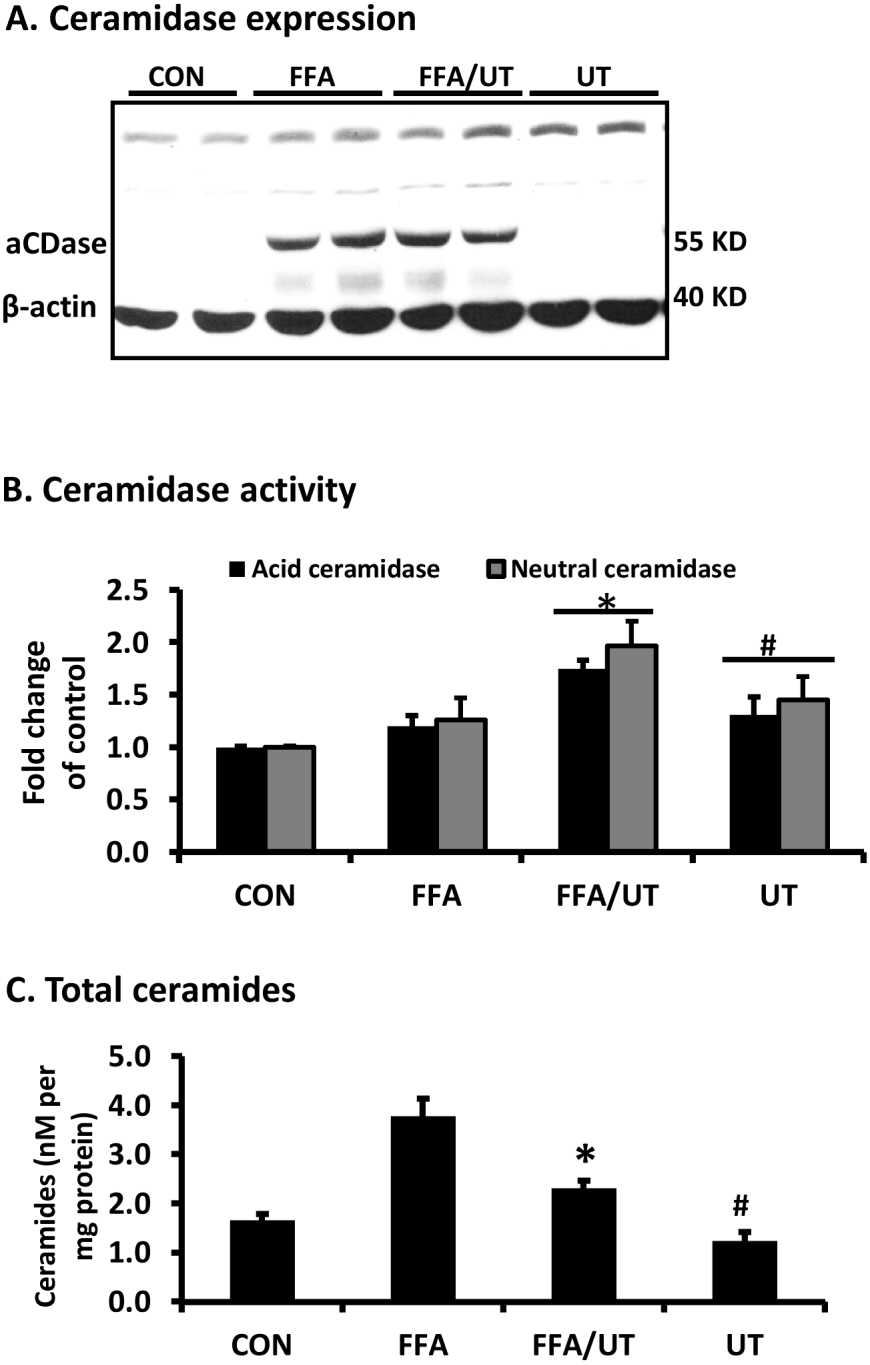
*Urtica dioica* extract does not affect ceramidase expression but enhances ceramidase activity and reduces ceramide levels in adipocytes. Fully differentiated 3T3-L1 adipocytes were incubated with palmitate (800 uM) and UT extract (5ug/ml) for 72 hours and **(A)** Cell lysates were used for western blot analysis. **(B)** Acid ceramidase activity was determined by fluorometric quantification of the fluorescent sphingosine generated in the cells after 24 hours incubation with C12 NBD ceramide. Neutral ceramidase was similarly determined after incubation with C18 NBD ceramide. **(C)** Total ceramides were quantified by tandem mass spectrometry (LCMS/MS). Each panel represents an experiment independently repeated three times on different batches of adipocytes. Treatments were compared by ANOVA followed by Tukeys test of multiple comparisons. *denotes significant difference between the FFA and FFA + UT treatments (P<0.05). ^**#**^denotes significant difference between the control treatments and UT only treatment (P<0.05).

Exposure of adipocytes to FFAs significantly increased the expression of acid ceramidase and neutral ceramidase protein in adipocytes in a dose dependent manner (Fig 2A). However, the activity of the enzymes was not significantly changed by FFA treatment (Fig 2B). Ceramidase expression was not affected by UT treatment alone (Fig 3A). However, treatment with UT extract in the presence of excess FFAs significantly increased ceramidase activity (P<0.05; Fig 3B). As expected, treatment with FFAs (800 uM) significantly increased the accumulation of total ceramides in cells. Co-incubation of FFAs and UT at 5ug/ml significantly reduced ceramide accumulation compared to FFA treated cells (P<0.05; Fig 3C). In addition, UT treatment alone reduced ceramides compared to the vehicle treatment (P<0.05; Fig 3C).

### Effects of FFAs and *Urtica dioica* extract following adiponectin knock down

**Fig 4:**
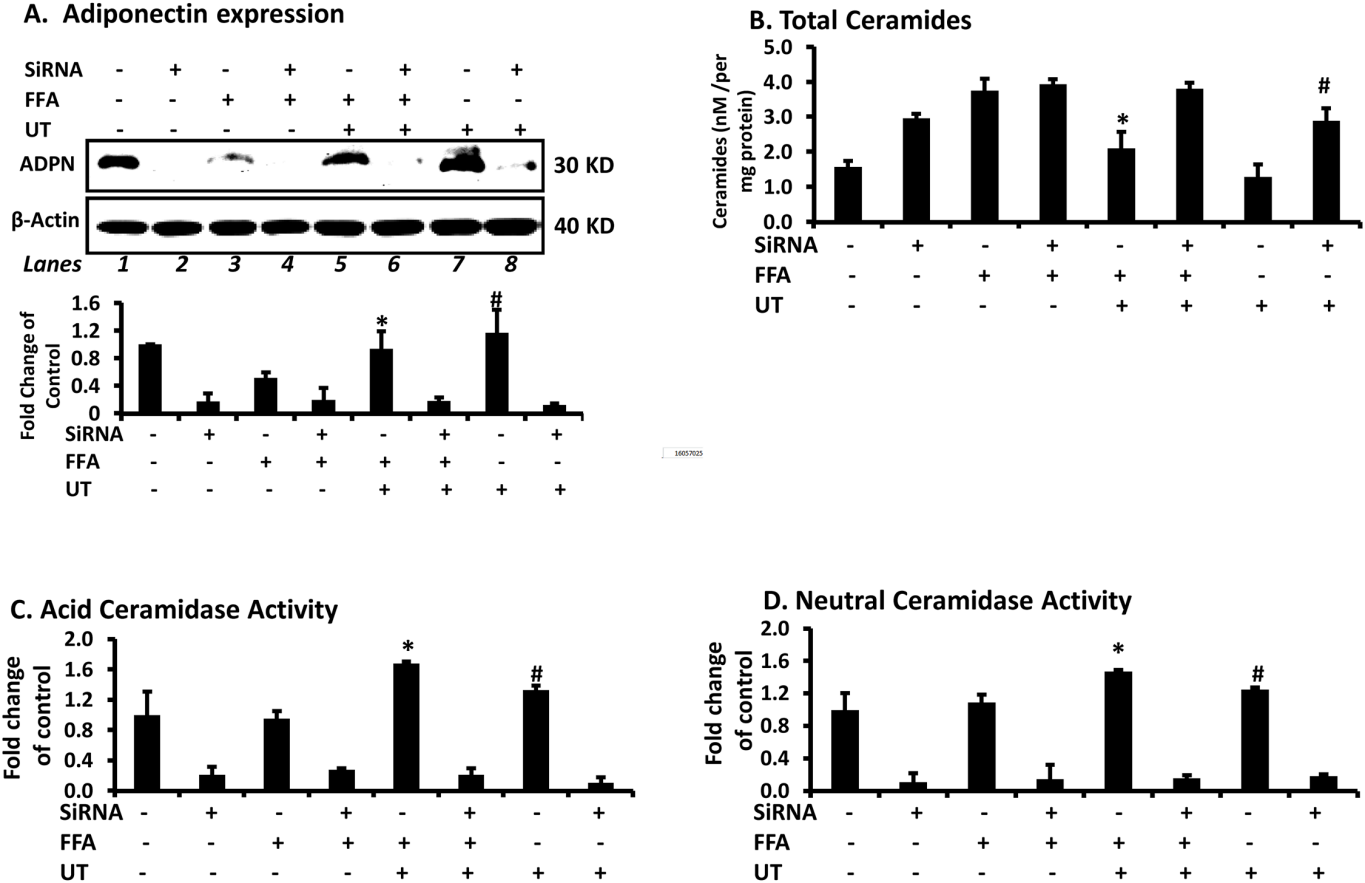
Adiponectin expression is required for *Urtica dioica* L. to enhance ceramidase activity and decrease ceramide accumulation. **(A)** Fully differentiated 3T3-L1 adipocytes were transfected with either a non-targeting siRNA or adiponectin siRNA and treated 48 hours later with palmitate (800uM) and/or UT extract (5ug/ml) for 72 hours. Cell lysates were used for western blot analysis to examine adiponectin expression. **(B)** Total ceramides were quantified by tandem mass spectrometry (LCMS/MS). **(C)** Acid ceramidase activity was determined by fluorometric quantification of the fluorescent sphingosine generated in the cells after 24 hours incubation with C12 NBD ceramide. **(D)** Neutral ceramidase was similarly determined after incubation with C18 NBD ceramide. Each panel represents an experiment independently repeated three times on different batches of adipocytes. Treatments were compared by ANOVA followed by Tukeys test of multiple comparisons.*denotes significant difference between the FFA and FFA + UT treatments (P<0.05) in adipocytes transfected with non-targeting siRNA (P<0.05). ^**#**^denotes significant difference between the control treatments and UT only treatment in adipocytes transfected with non-targeting siRNA (P<0.05).

To assess the potential mechanisms involved in the observed favorable effects of UT, we used siRNA to knock down adiponectin expression by approximately 80–90% in 3T3-L1 adipocytes (Fig 4A). In adipocytes transfected with non-target siRNA, FFAs decreased adiponectin expression and UT partially rescued the decrease in adiponectin induced by FFAs (Fig 4A, lane 3 vs. lane 5, P<0.05). In adipocytes with adiponectin knockdown, UT extract had no effect on adiponectin expression (Fig 4A, lane 4 vs. lane 6 and lane 2 vs. lane 8; P = ns). In cells transfected with non-targeting siRNA, UT reduced ceramide quantities in both control cells and those treated with FFAs (P<0.05; Fig 4B). In cells with adiponectin knock down, UT extract had no effects on FFA induced ceramide accumulation (Fig 4B). In cells transfected with the non-targeting siRNA, UT enhanced acid ceramidase activity and neutral ceramidase activity both in presence or absence of FFAs (P<0.05; Fig 4C and 4D). However, UT did not affect ceramidase activity in adipocytes that had highly reduced adiponectin expression due to siRNA knockdown.

### Effects of FFAs and *Urtica dioica* extract on Akt phosphorylation

**Fig 5:**
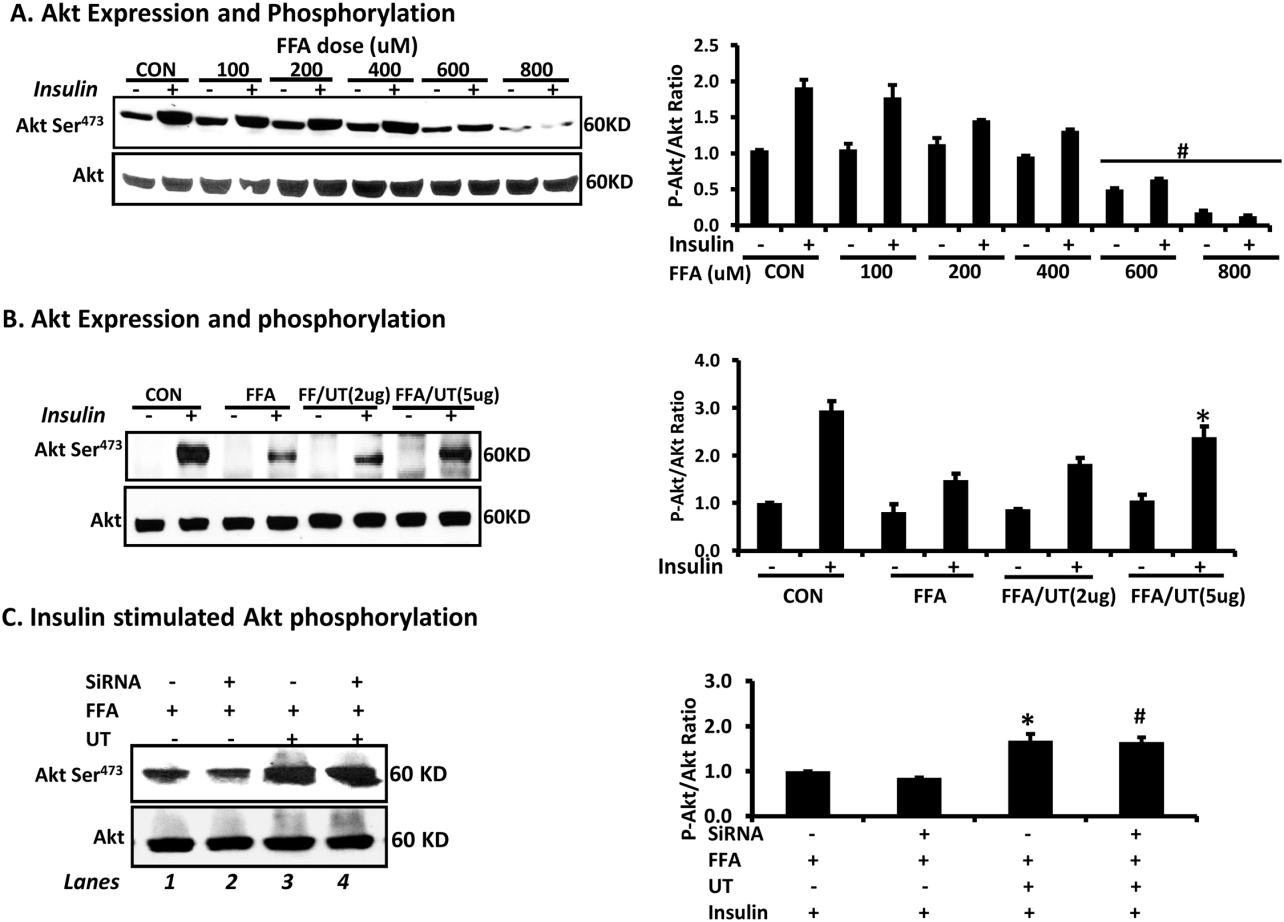
*Urtica dioica* extract enhances Akt phosphorylation in FFA treated cells in absence of Adiponectin. Fully differentiated 3T3-L1 adipocytes were incubated with indicated doses of **(A)** palmitate or **(B)** palmitate (800uM) and UT. Prior to harvest, adipocytes were stimulated with vehicle or 100 nM insulin for 8 minutes before collection in lysis buffer. Cell lysates were used for western blot analysis to examine Akt serine phosphorylation. Each panel represents an experiment independently repeated three times on different batches of adipocytes. Treatments were compared by ANOVA followed by the Tukeys test of multiple comparisons. ^#^denotes significant difference between the control and FFA only or UT only treatments (P<0.05). *denotes significant difference between the FFA and FFA + UT treatments (P<0.05). **(C)** Fully differentiated 3T3-L1 adipocytes were transfected with either a non-targeting siRNA or adiponectin siRNA and treated 48 hours later with palmitate (800uM) and/or UT extract (5ug/ml) for 72 hours. Prior to harvest, adipocytes were stimulated with 100 nM insulin for 8 minutes before collection in lysis buffer. Cell lysates were used for western blot analysis to examine Akt serine phosphorylation. Each panel represents an experiment independently repeated three times on different batches of adipocytes. Treatments were compared by ANOVA followed by the Tukeys test of multiple comparisons. *denotes significant difference between the FFA and FFA + UT treatments in adipocytes transfected with non-targeting siRNA. ^**#**^denotes significant difference between the FFA and FFA + UT treatments in adipocytes with adiponectin knockdown (P<0.05).

Acute insulin stimulation resulted in robust Akt serine phosphorylation that was diminished with increasing concentration of FFAs (P<0.05; Fig 5A). Co-incubation with FFA and 5ug/ml UT significantly attenuated the ability of FFAs to reduce Akt serine phosphorylation (P<0.05; Fig 5B). We also examined the ability of UT to effect Akt serine phosphorylation in the presence and absence of adiponectin (Fig 5C, lane 1 vs. lane 3 and lane 2 vs. lane 4; P<0.05) where adiponectin levels were depleted via siRNA. The efficacy of the siRNA adiponectin knockdown is shown in Fig 4A. These results show that the ability of UT to rescue the effect of FFAs on reducing Akt serine phosphorylation is not effected by adiponectin levels in adipocytes (Fig 5C).

## Discussion

Although UT is used in several cultures, and diet supplements are widely available in the United States, not much is known about the efficacy of this botanical in improving health conditions. In rodents, glucose tolerance tests indicate that UT lowers blood sugar, enhances islet insulin secretion and reduces inflammation [7–11]. However, there are minimal studies of UT in humans and its mechanism(s) of action are largely unknown. In the current study, we evaluated the effects of UT on adiponectin expression, ceramide accumulation and Akt serine phosphorylation in adipocytes. As a model of metabolic dysfunction, we utilized 3T3-L1 adipocytes treated with excess FFAs [12]. We show that UT attenuated the ability of FFAs to promote ceramide accumulation. In addition, UT attenuated the ability of FFAs to reduce adiponectin expression and Akt serine phosphorylation. We further show that UT impacts these positive effects by enhancing the enzyme activity of ceramidases that catabolize ceramides. Our novel observations demonstrate that the ability of UT to modulate ceramidase activity in adipocytes is dependent on the presence of adiponectin, but UT enhances insulin stimulated Akt serine phosphorylation even in the absence of adiponectin (Fig 5C).

The connections between obesity and metabolic diseases are well established. Obesity leads to elevated levels of circulating FFAs that are converted into metabolites including ceramides that induce insulin resistance [17, 18]. Excess FFAs also impact the secretory function of adipose tissue by changing the profile of adipokines and chemokines to favor secreted factors that are associated with insulin resistance. In particular, FFAs decrease adiponectin while enhancing the expression of TNFα, IL6, and RBP4 [12]. Excess FFAs, particularly palmitate, also stimulate lysosomal degradation of newly synthesized adiponectin [12]. Our study provides evidence that UT favorably modulates the function of fat cells by partially rescuing FFA induced reduction of adiponectin expression and secretion (Fig 1). These results suggest that UT treatment is metabolically beneficial since adiponectin can improve whole body glucose tolerance [19].

Ceramides may be formed through several pathways but can only be degraded through the activity of ceramidases, a family of enzymes that degrade them into sphingosine and free fatty acids [20]. Ceramide levels are carefully regulated in normal cells and numerous studies have shown that their accumulation induces insulin resistance [1–3, 17, 18, 20–24]. Agents that prevent ceramide accumulation or enhance ceramide degradation mitigate the inhibitory effects of saturated FFAs on insulin signaling [2,3,17,18]. For example, the overexpression of acid ceramidase in FFA treated C2C12 myotubes has been shown to prevent the effects of FFAs on insulin signaling by lowering ceramide levels [17]. Our data shows that FFAs increased ceramidase expression with a massive increase observed at 600 uM dose (Fig 3). Although FFA increased ceramidase expression, FFA did not alter ceramidase enzymatic activity. Hence, the levels of total ceramides, increased by over two fold (Fig 3C). There are three types of ceramidases with distinct protein sequences: acid, neutral and alkaline ceramidases, based on the pH at which their activity is optimal. Lysosomal acid ceramidase (pH optimum 4.0–5.0) prefers ceramide substrates with medium chain fatty acyl chains (6–16 carbons) and unsaturated fatty acids. In agreement with other reports [20], acid ceramidase (∼55 kDa) exhibited proteolytic cleaving into the α-subunit (13 kDa) and a β-subunit (43 kDa) (Figs 2A and 3A). Neutral ceramidase (pH spectrum of 7.0–9.0) is localized in mitochondria and prefers long-chain ceramides (16–18 carbons) [23, 24]. We evaluated both acid and neutral ceramidase expression and activity (Fig 2) because ceramide C16 is a preferred substrate for both enzymes. Three alkaline ceramidases (ACER1, ACER2, and ACER3) exist and reside in the endoplasmic reticulum [24]. We did not evaluate alkaline ceramidases (pH optimum ∼7.5–9.5) because the FFA used in this study (palmitic acid), primarily forms the saturated ceramide C16 that is not the preferred substrate of these ceramidases. ACER1 prefers very long chain unsaturated ceramides (≥24 carbons), ACER2 prefers very long chain saturated ceramides and ACER 3 does not hydrolyze saturated ceramides [23, 24].

Despite the FFA induced overexpression of ceramidases, adiponectin expression was reduced and ceramidase activity remained largely unchanged. Co-incubation with UT protected cells from the inhibitory effects of FFAs by enhancing adiponectin expression (Fig 1C), ceramidase activity (Fig 3B) and abating ceramide accumulation (Fig 3C). Our data suggested a connection between adiponectin levels and ceramidase activity. Knockdown of adiponectin negated the positive effects of UT on ceramidase activity and ceramide accumulation (Fig 4). In adipocytes transfected with the non-targeting siRNA, UT rescued the FFA induced reduction in adiponectin expression, increased ceramidase activity and prevented the accumulation of ceramides (Fig 4). These results indicate that ceramidases are active and effectively catabolize ceramide when adiponectin is present. Adiponectin knockdown caused ceramide accumulation even in adipocytes not treated with FFAs due to reduced ceramidase activity (Fig 4B). Our data corroborates that of Holland et al. [3] who showed that adiponectin exerts its beneficial metabolic effects *in vivo* by lowering hepatic ceramides. The modulation of ceramide versus sphingosine levels by ceramidases has physiological consequences. Because UT enhances the activity of ceramidases, it is likely that this botanical may also have significant effect on the sphingolipid metabolic pathway. Owing to their ability to regulate ceramide, sphingosine and sphingosine-1-phosphate, ceramidases control cellular processes and signal transduction pathways mediated by these lipids [21–24].

An inhibitory effect of ceramides on insulin signaling results from their ability to block the phosphorylation and activation of Akt a serine/threonine kinase that mediates glucose uptake and anabolic metabolism [1–3, 18]. Inhibiting ceramidase activity inactivates Akt, consistent with its ability to induce ceramide accumulation [18]. Our data demonstrates that FFA induced dysregulation of insulin signaling, as judged by Akt activation, occurs in a dose dependent manner. However, the presence of UT restored the ability of insulin to induce Akt phosphorylation in the presence of excess FFAs (Fig 5). A significant finding of our study was that UT enhanced Akt serine phosphorylation even in the absence of adiponectin (Fig 5C). This suggests that, in addition to ceramidase activation and reduction in ceramide accumulation, UT employs adiponectin independent mechanisms in enhancing Akt phosphorylation.

Our data clearly shows a relationship between the effects of UT and adiponectin expression and ceramidase activity. However, a limitation of our study is that it is unclear how adiponectin action impacts ceramidase activation in adipocytes. It is possible that adiponectin influences post-translational modification, proteolytic processing or subcellular trafficking including increased binding of ceramidase to lipid vesicles. Examining these parameters and identification of the active components of UT extract are important next steps for determining its potential as a metabolically favorable botanical supplement for obesity induced insulin resistance. We conclude that the effect of UT on ceramide metabolism via adiponectin expression is one of the mechanism(s) by which this botanical mediates insulin sensitivity.

## Supporting Information

S1 Fig Whole uncropped western blots for Fig 1A–1D. (TIF)

S2 Fig Whole uncropped western blots for Fig 2A. (TIF)

S3 Fig Whole uncropped western blot for Fig 3A. (TIF)

S4 Fig Whole uncropped western blot for Fig 4A. (TIF)

S5 Fig Whole uncropped western blots Fig 5A–5C. (TIF)

## References

[1] Obanda DN, Hernandez A, Ribnicky D, Yu Y, Zhang XH, Wang ZQ, Cefalu WT. Bioactives of *Artemisia dracunculus L*. mitigate the role of ceramides in attenuating insulin signaling in rat skeletal muscle cells. Diabetes. 2012; 61(3):597–605. 10.2337/db11-0396 22315320 PMC3282822

[2] Obanda DN, Ribnicky D, Raskin I, Cefalu WT. 2014 Bioactives of *Artemisia dracunculus L*. enhance insulin sensitivity by modulation of ceramide metabolism in rat skeletal muscle cells. Nutrition. 2014; 30 (7–8 Suppl):S59–66. 10.1016/j.nut.2014.03.006 24985108 PMC4082801

[3] Holland HL, Miller RA, Wang ZV, Sun K, Barth BM, Bui HH, et al The pleiotropic actions of adiponectin are initiated via receptor mediated activation of ceramidase activity. Nat Med. 2011; 17(1): 55–63. 21186369 10.1038/nm.2277 PMC3134999

[4] McLellan KCP, Wyne K, Villagomez ET, Hsueh WA. Therapeutic interventions to reduce the risk of progression from prediabetes to type 2 diabetes mellitus. Therapeutics and Clin. Risk Man. 2014; 10:173–188. 10.2147/TCRM.S39564 PMC3964168 24672242

[5] Ohio Agricultural Research and Development Center, Ohio State University. ‘Stinging Nettle.’ Available: http://www.oardc.ohio-state.edu/weedguide/singlerecord.asp?id=210

[6] Mehri A, Hasani-Ranjbar S, Larijani B, Abdollahi M. A systematic review of efficacy and safety of *Urtica dioica* in the treatment of diabetes. Int. J. Pharmacology. 2011; 7(2):161–170.

[7] Bnouham M, Merhfour F-Z, Ziyyat A, Mekhfi H, Aziz M, Legssyer A. Antihyperglycemic activity of the aqueous extract of *Urtica dioica*. Fitoterapia. 2003; 74(7–8): 677–681. 14630172 10.1016/s0367-326x(03)00182-5

[8] Golalipour MJ, Khori V. The protective activity of *Urtica dioica* leaves on blood glucose concentration and beta-cells in streptozotocin-diabetic rats. Pakistan J. Biol. Sci. 2007; 10(8):1200–1204. 10.3923/pjbs.2007.1200.1204 19069917

[9] University of Maryland Medical Center. ‘Stinging nettle’. Available: http://umm.edu/health/medical/altmed/herb/stinging-nettle#ixzz3NJw8kBGk

[10] Farzami B, Ahmadvand D, Vardasbi S, Majin FJ, Khaghani S. Induction of insulin secretion by a component of *Urtica dioica* leaf extract in perifused Islets of Langerhans and it’s *in vivo* effects in normal and streptozotocin diabetic rats J. Ethnopharmacology. 2003; 89(1):47–53. 14522431 10.1016/s0378-8741(03)00220-4

[11] Riehemann K, Behnke B and Schulze-Osthoff K. Plant extracts from stinging nettle (*Urtica dioica*), an antirheumatic remedy, inhibit the proinflammatory transcription factor NF-κB. FEBS Letters. 1999; 442: 89–94. 9923611 10.1016/s0014-5793(98)01622-6

[12] Karki S, Chakrabarti P, Huang G, Wang H, Farmer SR, Kandror KV. The multi-level action of fatty acids on adiponectin production by fat cells. PLoS One. 2011; 6(11):e28146 10.1371/journal.pone.0028146 22140527 PMC3226650

[13] Green H and Kehinde O. An established preadipose cell line and its differentiation in culture. II. Factors affecting the adipose conversion. Cell. 1975; 5:19–27. 165899 10.1016/0092-8674(75)90087-2

[14] Richard AJ, Fuller S, Fedorcenco V, Beyl R, Burris TP, Mynatt R, et al *Artemisia scoparia* enhances adipocyte development and endocrine function *in vitro* and enhances insulin action *in vivo*. PLoS ONE. 2014; 9(6): e98897 10.1371/journal.pone.0098897 24915004 PMC4051605

[15] Laemmli UK. Cleavage of structural proteins during the assembly of the head of bacteriophage T4. *Nature*. 1970; 227(5259):680–685. 5432063 10.1038/227680a0

[16] Abramoff MD, Magalhaes PJ, Ram SJ. Image Processing with ImageJ. Biophotonics Int. 2004 11(7):36–42, 2004.

[17] Chavez JA, Holland WL, Bar J, Sandhoff K, Summers SA. Acid ceramidase overexpression prevents the inhibitory effects of saturated fatty acids on insulin signaling. J. Biol. Chem. 2005; 280:20148–20153. 15774472 10.1074/jbc.M412769200

[18] Stratford S, Hoehn KL, Liu F, Summers SA. Regulation of insulin action by ceramide: Dual mechanisms linking ceramide accumulation to the inhibition of Akt/protein kinase B. J. Biol. Chem. 2004; 279:36608–36615. 15220355 10.1074/jbc.M406499200

[19] Bergman RN, Mittelman SD. Central role of the adipocyte in insulin resistance. J. Basic Clin. Physiol. Pharmacol. 1998; 9:205–21 10212835 10.1515/jbcpp.1998.9.2-4.205

[20] Shtraizent N, Eliyahu E, Park J-H, He X, Shalgi R, Schuchman EH. Autoproteolytic cleavage and activation of human acid ceramidase. J. Biol. Chem. 2008; 283:11253–11259. 10.1074/jbc.M709166200 18281275 PMC2431059

[21] Zeidan YH, Jenkins RW, Korman JB, Norris JS, Hannun YA. Molecular Targeting of Acid Ceramidase: Implications to Cancer Therapy. Curr. Drug Targets. 2008; 9(8): 653–661. 18691012 10.2174/138945008785132358 PMC3402562

[22] Zhu Q, Shan X, Miao H, Lu Y, Xu J, You N, et al Acute activation of acid ceramidase affects cytokine-induced cytotoxicity in rat islet β-cells. FEBS Letters. 2009; 583:2136–41. 10.1016/j.febslet.2009.05.047 19497324

[23] Hannun YA, Luberto C, Argraves KM. Enzymes of sphingolipid metabolism: From modular to integrative signaling. Biochemistry. 2001; 40 (16): 4893–4903. 11305904 10.1021/bi002836k

[24] Mao C and Obeid LM. Ceramidases: regulators of cellular responses mediated by ceramide, sphingosine, and sphingosine-1-phosphate. Biochim. Biophys. Acta. 2008; 1781(9): 424–434. 10.1016/j.bbalip.2008.06.002 18619555 PMC2614331

