# Supplementary figures and images for: Stinging Nettle (Urtica dioica L.) Attenuates FFA Induced Ceramide Accumulation in 3T3-L1 Adipocytes in an Adiponectin Dependent Manner
Source: PLoS One. 2016 Mar 3;11(3):e0150252. doi: 10.1371/journal.pone.0150252 (PMC4777364; doi:10.1371/journal.pone.0150252)

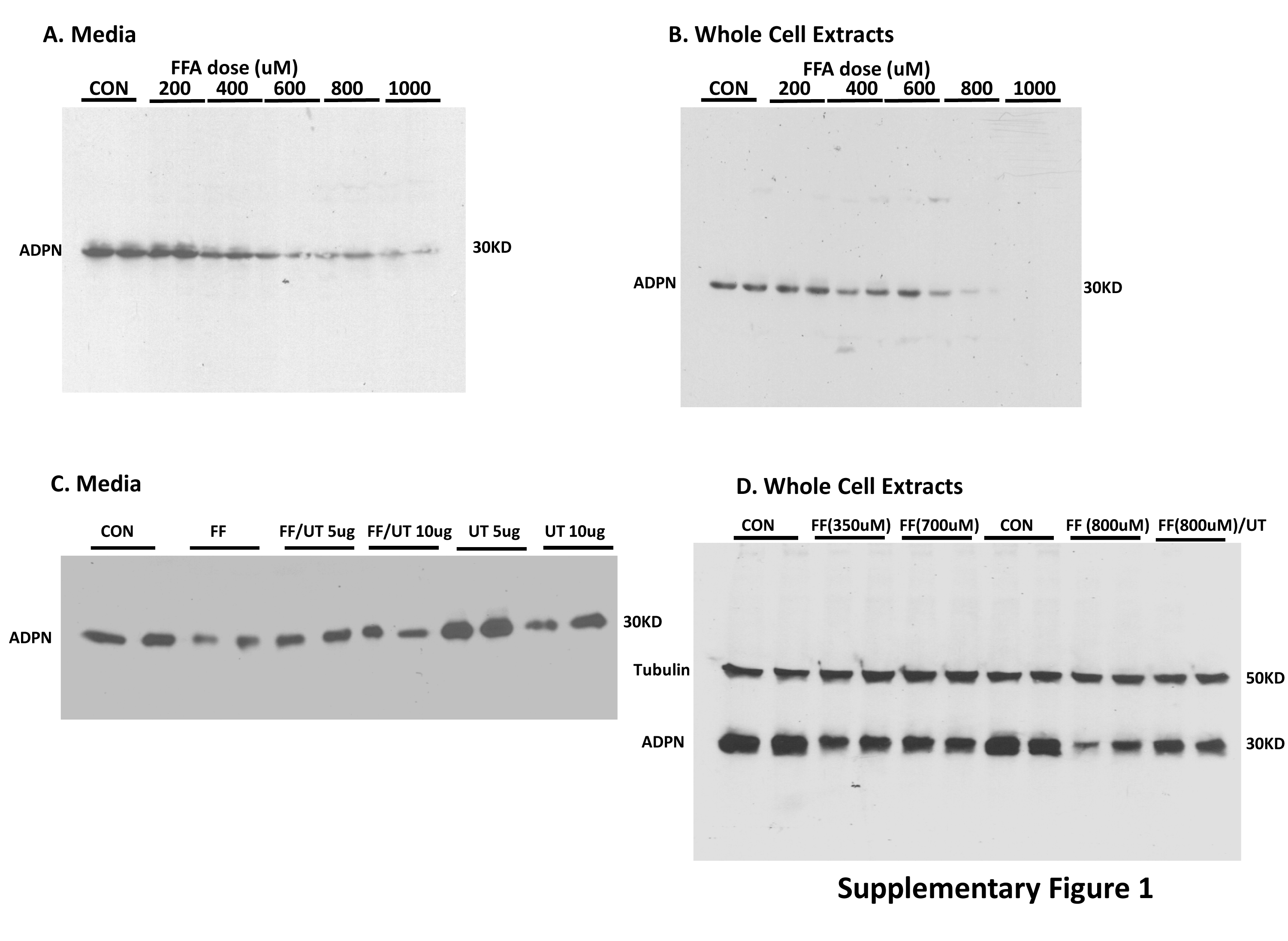

Supplement: S1 Fig — (TIF) [file pone.0150252.s001.tif]

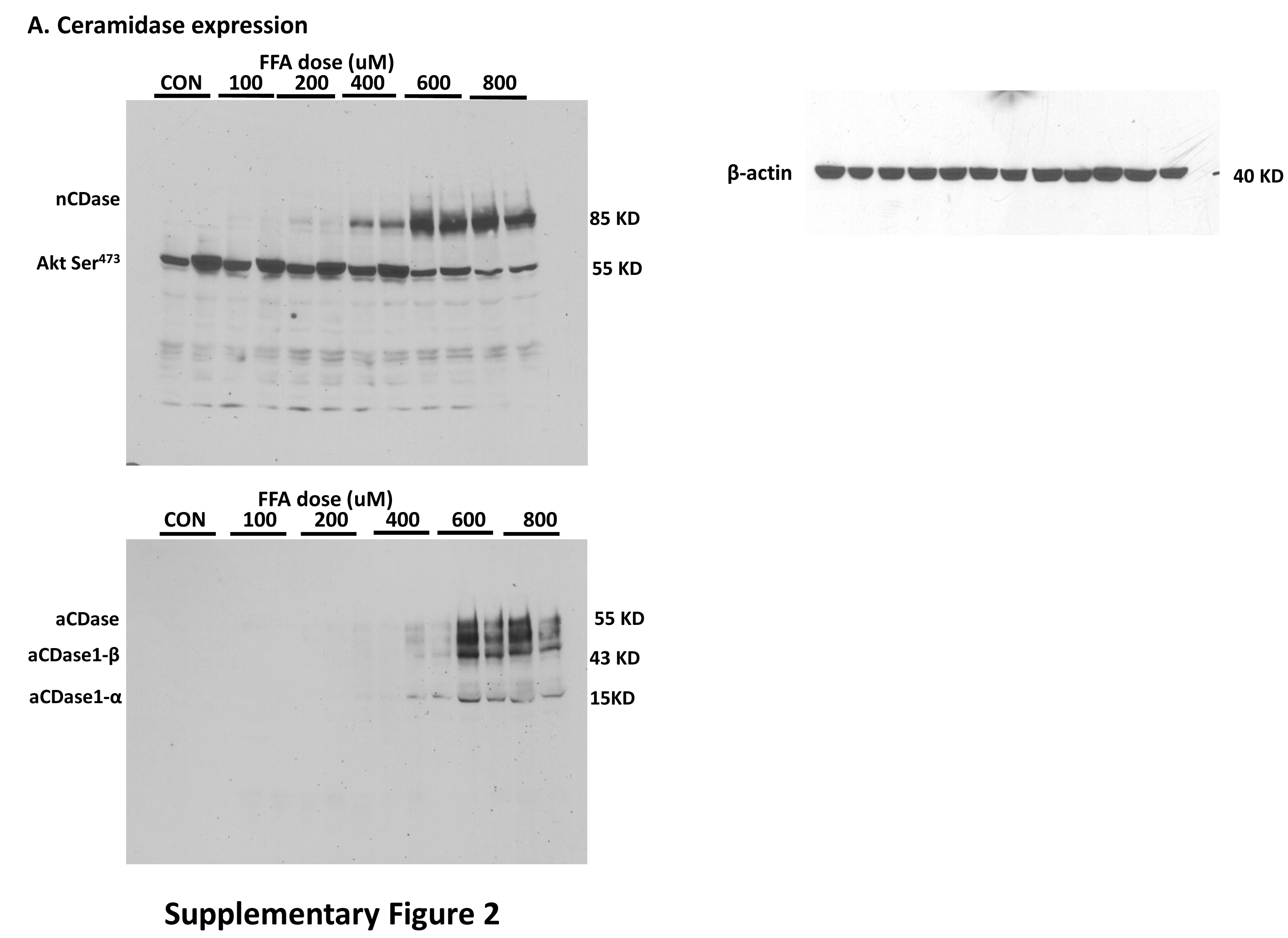

Supplement: S2 Fig — (TIF) [file pone.0150252.s002.tif]

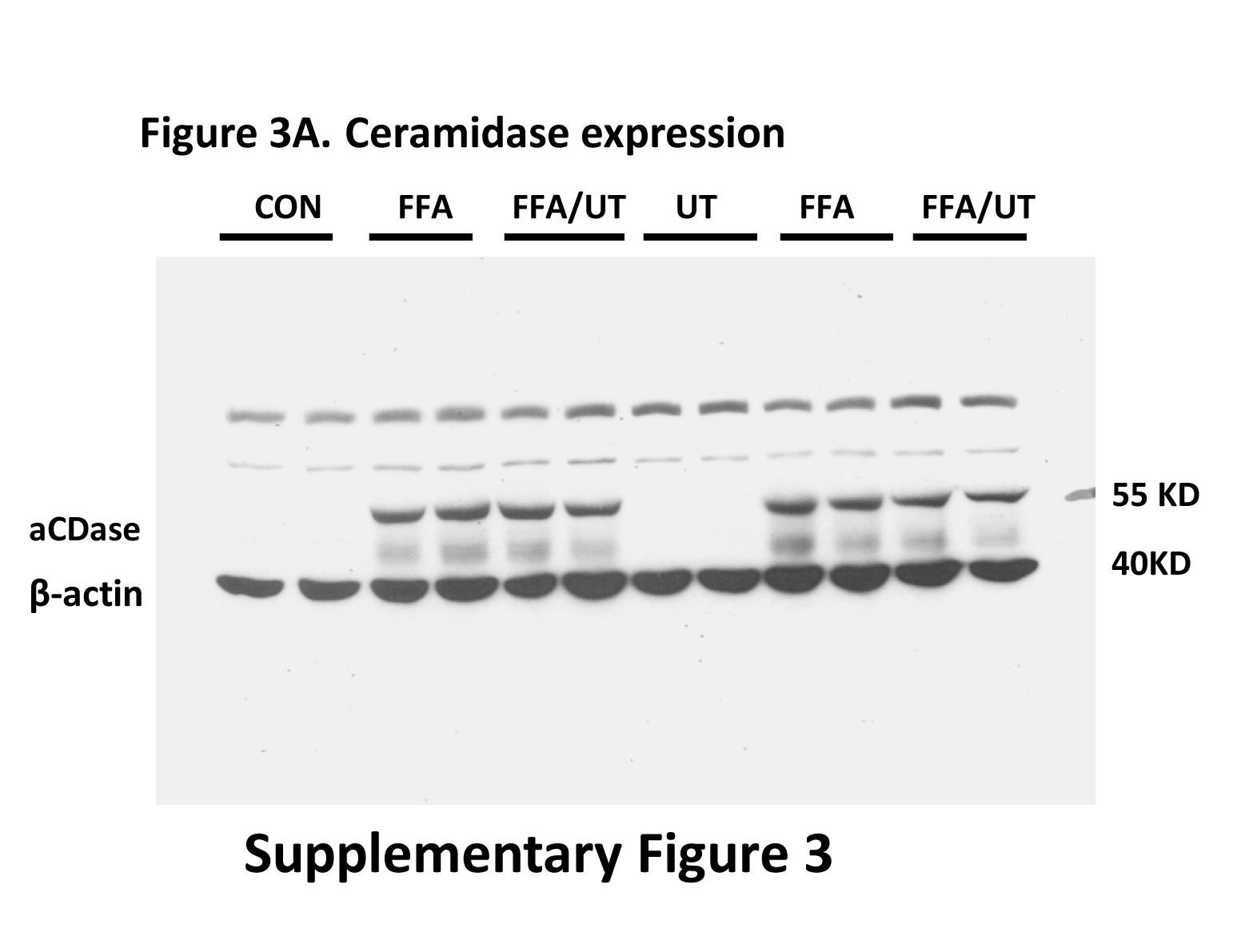

Supplement: S3 Fig — (TIF) [file pone.0150252.s003.tif]

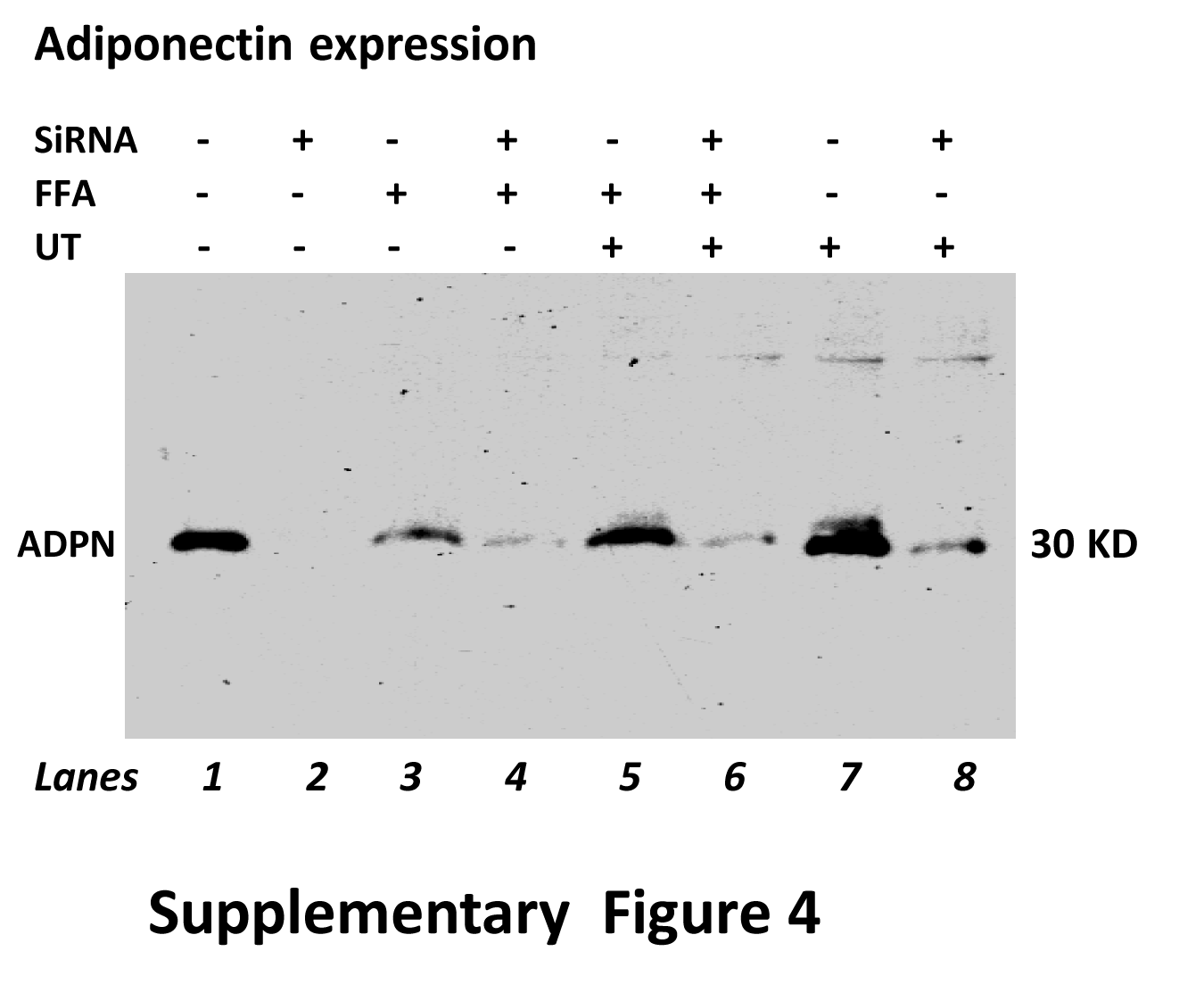

Supplement: S4 Fig — (TIF) [file pone.0150252.s004.tif]

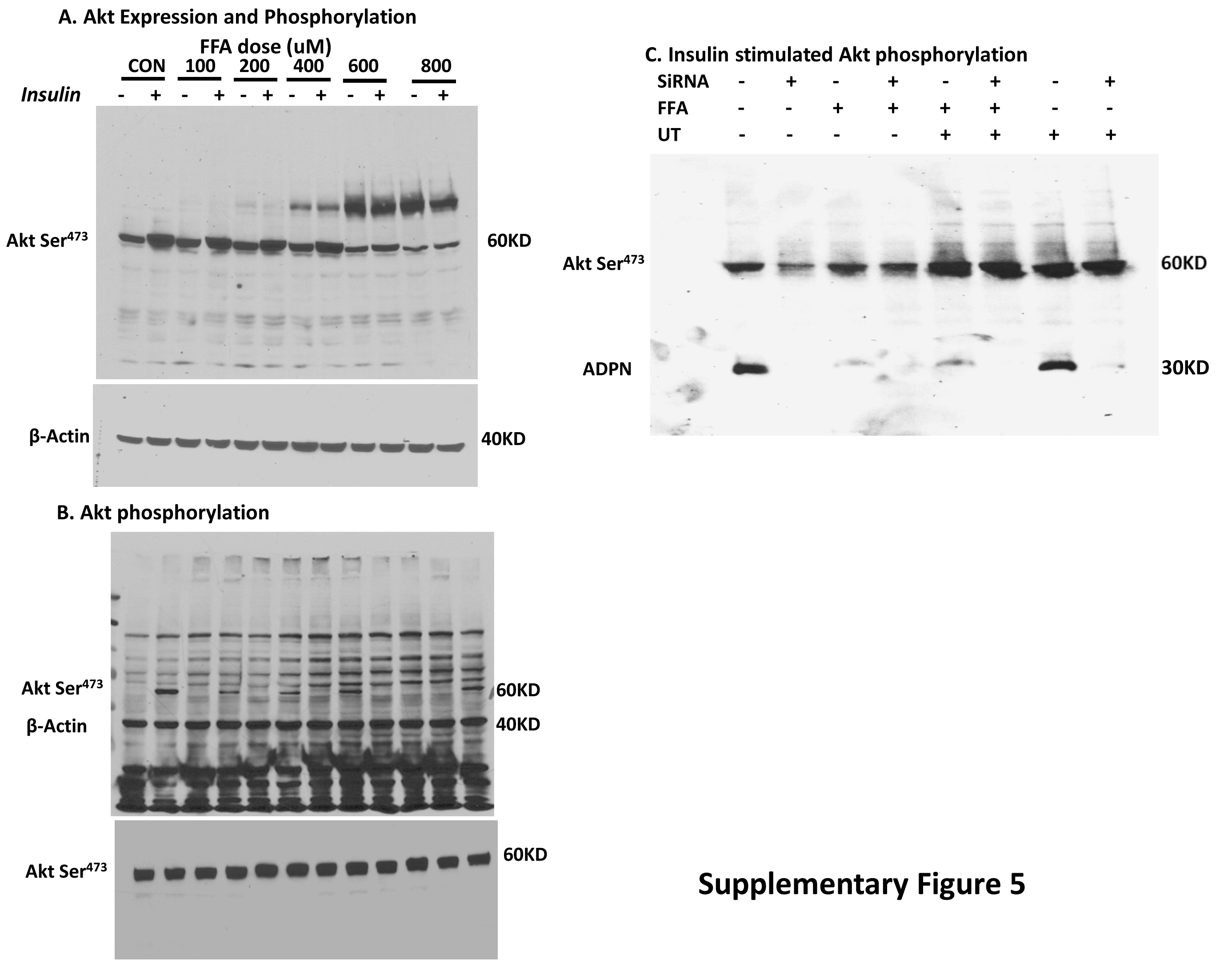

Supplement: S5 Fig — (TIF) [file pone.0150252.s005.tif]
